# Transmission of rabies through solid organ transplantation: a notable problem in China

**DOI:** 10.1186/s12879-018-3112-y

**Published:** 2018-06-14

**Authors:** Jian Zhang, Jun Lin, Ye Tian, Linlin Ma, Wen Sun, Lei Zhang, Yichen Zhu, Wei Qiu, Lujia Zhang

**Affiliations:** 1 Beijing Friendship Hospital, Capital Medical University, Beijing, China; 2 Beijing Key Laboratory of Tolerance Induction and Organ Protection in Transplantation, Beijing, China

**Keywords:** Donor, Encephalitis, Rabies, Transmission, Organ transplant

## Abstract

**Background:**

Due to the increasing number of DCD transplantations since 2015, the transmission of rabies through solid organ transplantation has become a notable problem in China and has attracted the attention of the public.

**Case presentation:**

From 2015 to 2017, four solid organ recipients in our centre were successively diagnosed with rabies that was considered to have been transmitted from two donors who died due to viral encephalitis of unknown cause and acute disseminated encephalomyelitis. The incubation periods were 44, 48, 158 and 303 days. The four patients had neurological symptoms associated with rabies and died. The survival times were 44, 34, 8 and 6 days. Another kidney transplant recipient received timely post-exposure prophylaxis and has remained asymptomatic.

**Conclusions:**

Organs should be discarded whenever rabies is confirmed or suspected, especially in cases diagnosed as encephalitis of unknown cause. It is important to establish a supervisory system to manage donor-derived infectious diseases. When rabies-infected donor organs are inadvertently transplanted, the recipients must receive post-exposure prophylaxis in a timely manner, which may be the only possible effective method to prevent the transmission of rabies.

## Summary of the article’s main point

Four recipients developed rabies that was considered to be transmitted via organ transplantation. Organs should be discarded whenever rabies is confirmed or suspected. A supervisory system is needed to manage donor-derived infectious diseases. Recipients should receive post-exposure prophylaxis in a timely manner when unexpected rabies-infected organs are transplanted.

## Background

Rabies is an acute and fatal viral encephalitis caused by viruses in the genus Lyssavirus, family Rhabdoviridae, which is acquired from infected animals via bites, scratches, or other exposure. From the point of entry, the virus is neurotropic, travelling quickly along the neural pathways into the central nervous system. After the brain is infected, the virus travels centrifugally to the peripheral and autonomic nervous systems, eventually migrating to the glands and organs [1].

The virus claims an estimated 59,000 (95% confidence interval (CI): 25000–159,000) human lives annually, mostly among underserved populations in Africa and Asia [2]. Over 95% of rabies deaths in humans result from virus transmission through bites by infected dogs [3]. In recent years, transmission via infected bats has become increasingly common. Transmission through infected animals including foxes, raccoons, skunks, jackals, or mongooses has been rarely reported. With the development of organ transplantation, the transmission of rabies through organ transplants has become a notable problem [4, 5]. In this study, we retrospectively reviewed four cases from our centre of probable transmission of rabies through organ transplants, which is now a common problem in China. Our aim is to summarize and analyse the clinical characteristics, treatment and prophylaxis of rabies transmitted through solid organ transplantation to guide clinical work.

## Case presentation

### Organ donors

**Fig. 1:**
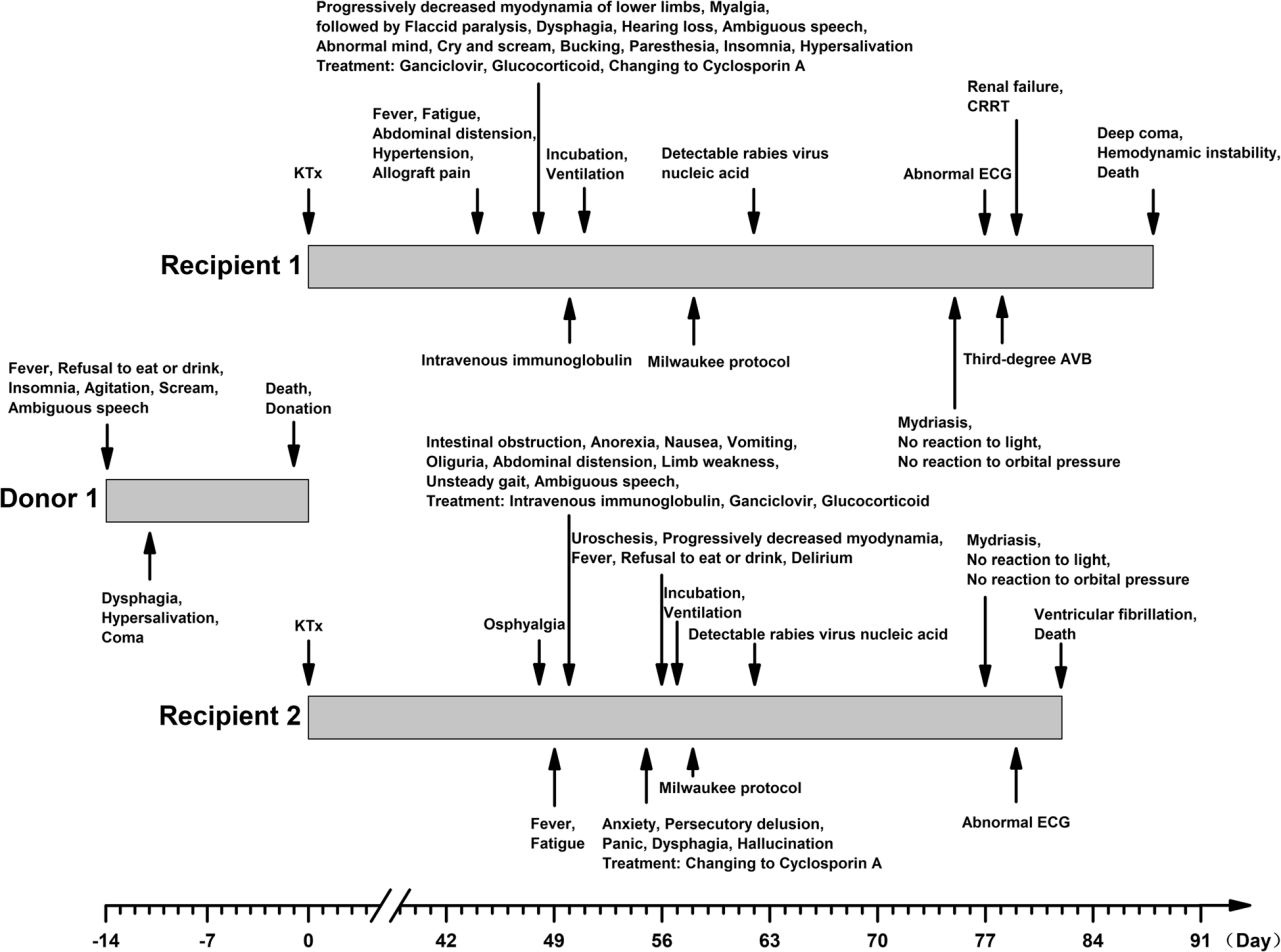
Transmission of rabies from donor 1 to recipient 1, 2

Donor 1 was a 6-year-old boy from Guangxi province in China. The initial symptoms on 13 May 2015 were fever of unknown cause with insomnia and refusal to eat or drink. He was sent to a local hospital due to subsequent agitation, screaming and incoherent speech. Three days later, he suffered from dysphagia and hypersalivation. His condition subsequently worsened despite treatment with ribavirin. Finally, he died on 26 May after receiving an initial diagnosis of viral encephalitis of unknown cause (Fig. 1). Cerebrospinal fluid (CSF) analysis revealed an opening pressure of 60 drops/min, transparent and limpid fluid, a glucose level of 4.7 mmol/L, and a protein level of 265 mg/L. Computed tomography (CT) revealed a slightly decreased density in the bilateral temporal lobes. Tests for HIV, hepatitis B, hepatitis C, and syphilis yielded negative results. Earlier, the donor had frequent contact with domestic dogs but no longer had this type of exposure since moving to live with his grandmother in another city. His family members denied either exposure to potentially rabid animals or history of rabies vaccinations. His kidneys and corneas were donated for transplantation.

**Fig. 2:**
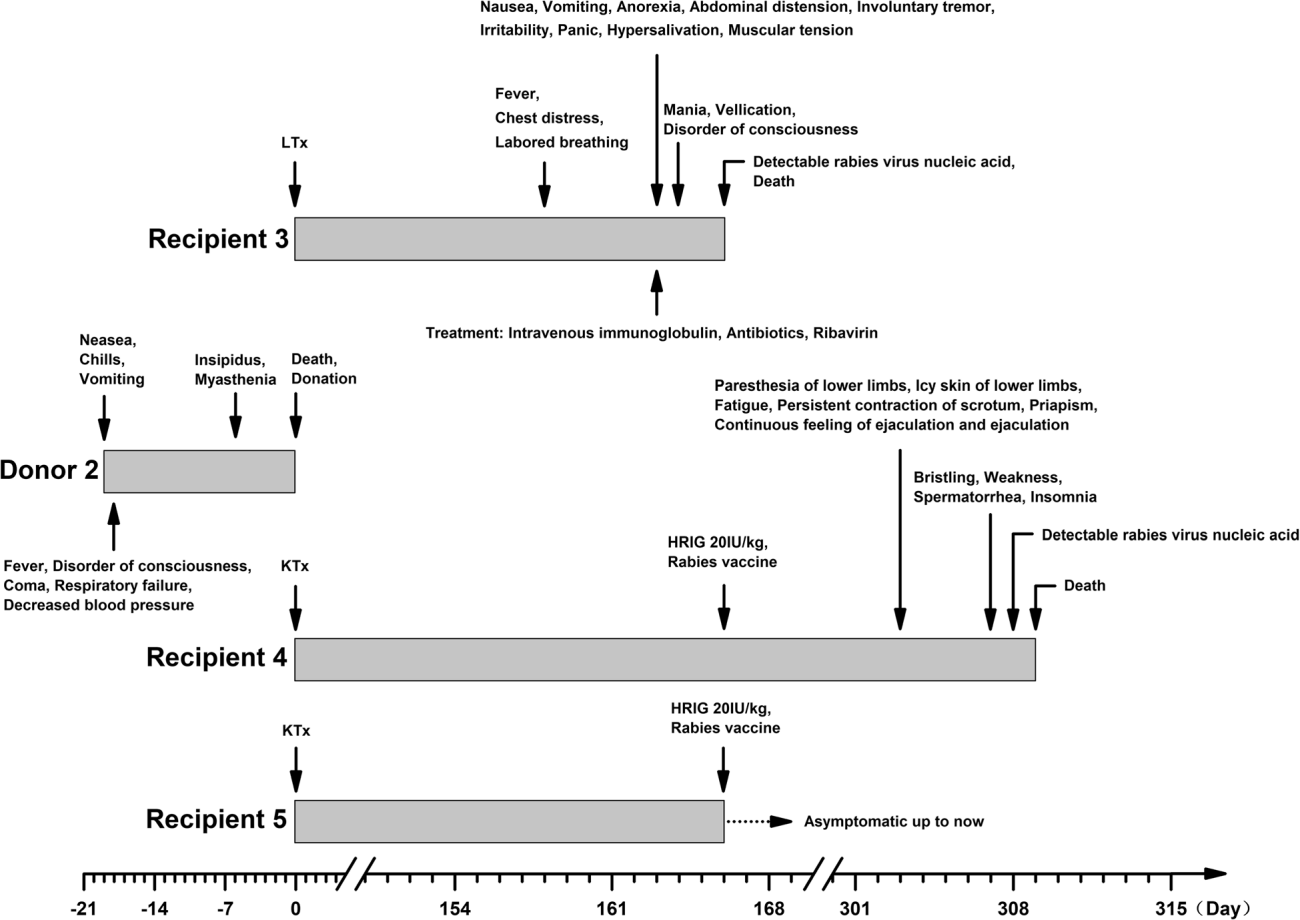
Transmission of rabies from donor 2 to recipient 3, 4

Donor 2 was an 11-year-old girl who lived in Hebei province in China. Her initial symptoms were nausea, chills and vomiting on 22 Sep 2016. One day later, her condition worsened, and she suffered from fever, disorder of consciousness, coma, respiratory failure and decreased blood pressure, followed by insipidus and myasthenia gravis on 5 Oct. She died on 11 Oct after an initial diagnosis of acute disseminated encephalomyelitis (Fig. 2). The results of CSF analysis were normal. Magnetic resonance imaging (MRI) revealed diffuse signal abnormalities throughout the brain and cervical spinal cord. Tests for HIV, hepatitis B, hepatitis C, syphilis, cytomegalovirus, Epstein Barr virus, coxsackie virus, herpes simplex virus, adenovirus and rubella virus yielded negative results. Her family members denied exposure to potentially rabid animals or history of rabies vaccinations. Her kidneys and liver were donated for transplantation.

### Transplant recipients

Recipient 1 was a 55-year-old male who received a kidney transplant from donor 1 on 27 May 2015. The allograft recovered successfully, and the immunosuppressive regimen included tacrolimus, mycophenolate sodium and prednisone. The patient initially became symptomatic on 10 Jul 2015 (Fig. 1). MRI revealed mottled signal abnormalities around the bilateral ventricles and deep white matter (low or equal signal on T1WI and high signal on T2WI and Flair), which indicated white matter demyelination. He was clinically diagnosed with rabies on 24 Jul according to the typical symptoms; this diagnosis was confirmed 4 days later by detectable rabies virus-specific nucleic acids in saliva, urine, and sputum samples. The patient died on 23 Aug. His family members denied exposure to potentially rabid animals or history of rabies vaccinations.

Recipient 2 was a 43-year-old male who received a kidney transplant from donor 1 on 27 May 2015. The allograft recovered successfully, and the immunosuppressive regimen included tacrolimus, mycophenolate sodium and prednisone. The patient initially became symptomatic on 14 Jul 2015 (Fig. 1). CT revealed a suspicious mottled low-density region in the right basal ganglia region. He was clinically diagnosed with rabies on 24 Jul according to the typical symptoms, which was confirmed 4 days later by detectable rabies virus-specific nucleic acids in saliva and urine samples. The patient died on 17 Aug. His family members denied exposure to potentially rabid animals or history of rabies vaccinations.

Recipient 3 was a 57-year-old female who received a liver transplant from donor 2 on 11 Oct 2016. The allograft recovered successfully, and the immunosuppressive regimen included tacrolimus, mycophenolate mofetil and methylprednisolone. The patient initially became symptomatic on 18 Mar 2017 (Fig. 2). CT revealed a suspicious mottled low-density region in the left basal ganglia region. She was clinically diagnosed with rabies on 24 Mar according to the typical symptoms, which was confirmed 2 days later by detectable rabies virus-specific nucleic acids in a saliva sample. The patient died on 26 Mar without invasive mechanical ventilation. Her family members disclosed that she had contact with a domestic pet half a year before transplantation but denied exposure to potentially rabid animals or history of rabies vaccinations.

Recipient 4 was a 50-year-old male who received a kidney transplant from donor 2 on 11 Oct 2016. The allograft recovered successfully, and the immunosuppressive regimen included tacrolimus, mycophenolate mofetil and prednisone. The patient received post-exposure prophylaxis (PEP) immediately after confirmation of rabies in deceased recipient 3 on 26 Mar 2017, which consisted of a 5-dose vaccination regimen (5 doses of purified chick embryo cell rabies vaccine, the first given directly after suspected exposure and subsequently on days 3, 7, 14, and 28) with 1 dose of rabies immunoglobulin (20 IU/kg). However, he still became initially symptomatic on 10 Aug 2017 (Fig. 2). He was clinically diagnosed with rabies on 14 Aug according to the typical symptoms, which was confirmed 1 day later by detectable rabies virus-specific nucleic acids in saliva, urine, and sputum samples. The patient died on 16 Aug without invasive mechanical ventilation. His family members denied exposure to potentially rabid animals or history of rabies vaccinations.

Recipient 5 was a 46-year-old male who received a kidney transplant from donor 2 on 11 Oct 2016. The allograft recovered successfully, and the immunosuppressive regimen included tacrolimus, mycophenolate sodium and prednisone. The patient received PEP immediately after confirmation of rabies in deceased recipient 3 on 26 Mar 2017, which consisted of a 5-dose vaccination regimen with 1 dose of rabies immunoglobulin. The recipient currently remains asymptomatic (Fig. 2). Unfortunately, the level of rabies-specific neutralizing antibody was not quantified.

### Laboratory findings

Rabies virus-specific nucleic acids were detectable in saliva, urine, and sputum samples from recipient 1 and were also detectable in saliva and urine samples from recipient 2. The same results were found in a saliva sample from recipient 3 and in saliva, urine, and sputum samples from recipient 4. According to diagnostic criteria in China [1], these recipients were laboratory-confirmed as positive for rabies.

## Discussion

With the development of organ transplantation, the transmission of rabies through organ transplants has gained increasing attention. Rabies transmitted through corneal transplantation was initially reported in 1978 by Houff [6], followed by several other reports. However, rabies transmitted through solid organ transplantation has rarely been reported for decades. In total, 11 cases of solid organ transplantation from rabies-infected donors were reported from different centres, among whom seven patients developed rabies [4, 5, 7, 8] (Table 1). All the patients died after developing rabies, yielding a mortality rate of 100%. In our centre, four of five patients developed rabies after organ transplantation from two rabies-infected donors. All the family members denied exposure to potentially rabid animals or history of rabies vaccinations. The two donors were initially diagnosed with viral encephalitis and acute disseminated encephalomyelitis, which were reclassified as rabies due to typical symptoms and confirmation of rabies in recipients who received the organs. Thus, the cause of rabies in the recipients was considered transmission through organ transplantation from infected donors.

**Table 1:** Occurrence of rabies transmitted through solid organ transplant

References	Countries	Solid organs	Developing rabies	Time
Srinivasan et al. [4]	USA	Liver	Yes	2004
		Kidney	Yes	2004
		Kidney	Yes	2004
Maier et al. [7]	Germany	Lung	Yes	2005
		Kidney	Yes	2005
		Pancreas-Kidney	Yes	2005
		Liver	No	N/A
Vora et al. [5, 8]	USA	Kidney	Yes	2013
		Kidney	No	N/A
		Liver	No	N/A
		Heart	No	N/A

The prevalence of rabies is high in China, with a total of 29,656 cases reported from 1996 to 2014, and it is mainly distributed in Southern China. The highest incidence is found in Guangxi, followed by Hunan, Guangdong, Guizhou and Jiangxi, accounting for 58.25% of all the cases in the country. The most common age of onset was 45–59 years (27.22%), followed by 0–14 years (22.07%). The most common occupation associated with rabies was farmer (65.46%), followed by student (14.82%) and scattered child (7.67%) [9]. However, although vaccination for dogs is obligatory, it is not extensively generalized, especially in rural areas or for stray dogs. Attention is mainly focused on prophylaxis after exposure to potentially rabid dogs instead of prophylaxis in dogs, which is a misunderstanding of proper prophylaxis against rabies. In this study, both donors seemed to be at high risk for exposure to rabies.

The incubation period of rabies is associated with toxicity and the distribution of nerves in the infected area and is commonly 2–3 months to less than 1 year in the general population. The survival time from onset of nervous system symptoms to death is 1–5 days without treatment [1]. Previous studies [4, 5, 7] found that the median incubation period of rabies transmitted through solid organ transplantation was 27 days (20–510 days), which is shorter than that observed in the general population. This finding is likely due to the transmission pathway, immunosuppression, or the numerous nerves in solid organs. The mean survival time from onset of symptoms to death was 26 ± 17 (9–60) days, which was most likely due to the use of treatment and life support. In this study, the incubation periods were 44, 48, 158 and 303 days, which is consistent with previous studies, except for recipients 3 and 4, which might be associated with low toxicity. The survival times were 44, 34, 8 and 6 days. Longer survival times may be associated with treatment, including intravenous immunoglobulin, high-dose corticosteroids, and the Milwaukee protocol. However, treatment cannot change the outcome of death.

Rabies is categorized into two types based on clinical symptoms, the manic type and paralytic rabies; the former is more common. However, in this study, paralytic rabies was more common than it was in the general population, with symptoms of weakness, fatigue, progressively decreased myodynamia, myalgia, paralysis, paraesthesia, or abdominal distension. These symptoms are similar to those of Guillain-Barre syndrome, which often results in misdiagnosis.

Although the mortality rate of rabies is extremely high, it can be prevented by active immunity and passive immunity after exposure. PEP includes vaccines and rabies immunoglobulin [10, 11]. According to the World Health Organization’s Expert Rabies Committee, a neutralizing antibody level of ≥0.5 International Units (IU)/mL of serum results in a positive protective antibody response to vaccination [3]. Notably, five recipients in total (including one recipient in our study) were asymptomatic, including two cases of liver transplantation, two cases of kidney transplantation and one case of heart transplantation. All these recipients received PEP immediately after confirmation of rabies in the donors and before the onset of symptoms. The level of neutralizing antibody after PEP was > 0.1 IU/mL in four recipients, three of whom had an antibody level ≥ 0.5 IU/mL, indicating a positive protective antibody response to vaccination. One liver transplant recipient received vaccination before transplantation. Although the antibody level of recipient 5 was unknown, it was considered to have reached the protective level. Vora reported long-term survival of three recipients [8]. In our study, recipient 5, who had not been vaccinated for rabies at the time of transplantation, achieved disease-free survival for 15 months, which was an unexpected outcome. One explanation for this finding was that he received a kidney without the rabies virus, given that the typical incubation period of the virus is less than 1 year. However, a long incubation period experienced by a recipient who eventually developed rabies has also been reported [5]. Furthermore, the other two recipients who received organs from the same donor developed rabies. Another possibility was that this recipient was exposed to the rabies virus with a low toxicity and was cleared prior to PEP. The last and most interesting possibility is that PEP administration for this recipient was effective. Thus, when rabies-infected donor organs are inadvertently transplanted, recipients must receive PEP administration in a timely manner to decrease the risk of developing rabies. However, PEP cannot change the outcome of death if the patient has already developed rabies, even if the neutralizing antibody is detectable.

Due to the increase in donation after citizen’s death (DCD) since 2015, the transmission of rabies through solid organ transplantation has become a notable problem in China and has widely attracted attention from the public. Due to the rapid progression of the disease and insufficient data on rabies, the misdiagnosis of rabies is common, which may result in an underestimation of the current data, especially in rural areas. Furthermore, restrictions on laboratory tests make it difficult to confirm the diagnosis of rabies within a sufficiently short time to determine whether to procure organs. In fact, donors, especially children, frequently die from infectious encephalitis of unknown cause. However, infectious encephalitis is not a contraindication for donation, which may lead to the potential risk of rabies transmission through organ transplantation. Thus, it is necessary for organ procurement organization (OPO) coordinators and clinical doctors to recognize rabies and improve the evaluation of donors, including obtaining a detailed medical history and evaluating the associated epidemiology of donors with a high risk of infectious disease, especially encephalitis of unknown cause. Serological testing and aetiological diagnosis are recommended for donors with a high risk of rabies or clinically suspected rabies. Organs should be discarded when rabies is confirmed or suspected, especially if the donor is diagnosed with encephalitis of unknown cause. Meanwhile, it is important to establish a supervisory system to manage donor-derived diseases.

## Conclusions

The transmission of rabies through solid organ transplantation is a life-threatening and notable problem in China. The mortality rate is extremely high after the development of rabies. Serological testing and aetiological diagnosis are recommended for donors with a high risk of rabies or clinically suspected rabies. Organs should be discarded when rabies is confirmed or suspected, especially if the donor is diagnosed with encephalitis of unknown cause. It is important to establish a supervisory system to manage donor-derived infectious disease. When rabies-infected donor organs are inadvertently transplanted, recipients must receive PEP in a timely manner, which may be the only possible and effective method to prevent transmission of rabies.

### Limitations

This was a retrospective study. Data regarding many related factors were incomplete or unavailable.

## Abbreviations

CSF: Cerebrospinal fluid
CT: Computed tomography
DCD: Donation after citizen’s death
MRI: Magnetic resonance imaging
OPO: Organ procurement organization
PEP: Post-exposure prophylaxis
